# Dysregulation of Inositol Polyphosphate 5-Phosphatase OCRL in Alzheimer’s Disease: Implications for Autophagy Dysfunction

**DOI:** 10.3390/ijms26125827

**Published:** 2025-06-18

**Authors:** Kunie Ando, May Thazin Htut, Eugenia Maria Antonelli, Andreea-Claudia Kosa, Lidia Lopez-Gutierrez, Carolina Quintanilla-Sánchez, Emmanuel Aydin, Emilie Doeraene, Siranjeevi Nagaraj, Ana Raquel Ramos, Katia Coulonval, Pierre P. Roger, Jean-Pierre Brion, Karelle Leroy

**Affiliations:** 1 Alzheimer and Other Tauopathies Research Group, ULB Neuroscience Institute (UNI), ULB Center for Diabetes Research (UCDR), Faculty of Medicine, Université Libre de Bruxelles, 808 Route de Lennik, 1070 Brussels, Belgiumandreea-claudia.kosa@ulb.be (A.-C.K.); lidia.lopez.gutierrez@ulb.be (L.L.-G.); carolina.quintanilla.sanchez@ulb.be (C.Q.-S.); emmanuel.aydin@ulb.be (E.A.); emilie.doeraene@ulb.be (E.D.); siranjeevi.nagaraj@ulb.be (S.N.); jean-pierre.brion@ulb.be (J.-P.B.); 2 IRIBHM-Jacques E. Dumont, Campus Erasme, Université Libre de Bruxelles, 1070 Brussels, Belgiumkcoulonv@ulb.ac.be (K.C.); pierre.roger@ulb.be (P.P.R.)

**Keywords:** Alzheimer’s disease, OCRL, Beclin1, autophagy, phosphatidylinositol, tau, amyloid ß, pTau

## Abstract

Autophagy is impaired in Alzheimer’s disease (AD), particularly at the stage of autophagosome–lysosome fusion. Recent studies suggest that the inositol polyphosphate 5-phosphatase OCRL (Lowe oculocerebrorenal syndrome protein) is involved in this fusion process; however, its role in AD pathophysiology remains largely unclear. In this study, we investigated the localization and expression of OCRL in post-mortem AD brains and in a 5XFAD transgenic mouse model. While *OCRL* RNA levels were not significantly altered, OCRL protein was markedly reduced in the RIPA-soluble fraction and positively correlated with the autophagy marker Beclin1. Immunohistochemical analysis revealed OCRL immunoreactivity in neuronal cytoplasm, granulovacuolar degeneration bodies, and plaque-associated dystrophic neurites in AD brains. Furthermore, OCRL overexpression in a FRET-based tau biosensor cell model significantly reduced the tau-seeding-induced FRET signal. These findings suggest that OCRL dysregulation may contribute to autophagic deficits and the progression of tau pathology in AD.

## 1. Introduction

Alzheimer’s disease (AD) is characterized by two hallmark neuropathological lesions: amyloid plaques and neurofibrillary tangles (NFTs). Amyloid plaques are composed of amyloid-β (Aβ) peptides, which result from the proteolytic processing of the amyloid precursor protein (APP) [1]. NFTs consist of hyperphosphorylated and aggregated paired helical filament (PHF) tau [2].

In healthy conditions, phosphoinositide (PI) levels are tightly regulated by PI kinases and PI phosphatases [3]. However, PI homeostasis is disrupted in AD brains compared to healthy controls [4,5,6].

Lowe oculocerebrorenal syndrome protein (OCRL) is a ubiquitously expressed PI 5-phosphatase. Loss-of-function mutations in OCRL cause Lowe syndrome, a rare X-linked recessive disorder characterized by renal tubular dysfunction, developmental delay, intellectual disability, and congenital cataracts [7]. OCRL is one of ten known PI 5-phosphatases in the human genome [8]. It regulates levels of the second messenger PI(4,5)P_2_ by dephosphorylating it at the 5-position to generate PI4P [3,9]. While OCRL is primarily associated with Lowe syndrome, it also plays a crucial role in autophagy, particularly in autophagosome–lysosome fusion, by triggering a lysosomal response under its control [10].

OCRL is a cytosolic protein that localizes to multiple subcellular compartments, including clathrin-coated vesicles, early endosomes, the trans-Golgi network (TGN), and lysosomal membranes. In healthy kidney proximal tubule cells, OCRL is present at these sites, but it is recruited to lysosomes in response to autophagosome–lysosome fusion in an AP2- and clathrin-dependent manner [10]. OCRL deficiency disrupts lysosomal dynamics and proteolytic activity [10,11].

Autophagy is impaired in AD, and several autophagy-related proteins, such as Beclin1, are downregulated in AD brains [12]. However, the expression levels, solubility, and potential pathological associations of OCRL in post-mortem AD brain tissue remain poorly understood.

In this study, we investigated the localization and regulation of OCRL in AD brains. We found that OCRL accumulates in plaque-associated dystrophic neurites and is depleted from the RIPA-soluble fraction of AD brain lysates, in parallel with a reduction in the autophagy marker Beclin1. Furthermore, OCRL overexpression in HEK tau biosensor cells attenuated tau seeding, suggesting a potential protective role for OCRL. Together, our findings highlight significant dysregulation of OCRL in AD and implicate it in autophagy dysfunction and tau pathology.

## 2. Results

### 2.1. OCRL Protein Accumulates in Dystrophic Neurites in AD Brains

We first assessed the localization of OCRL in post-mortem brain tissues from control and AD cases. The specificity of the rabbit polyclonal anti-OCRL antibody (Proteintech, fisher scientific, Merelbeke, Belgium, 17695-1-AP) used in this study has been validated in several independent reports, including studies employing OCRL-knockout or -knockdown cells [13,14,15], as well as tissues from patients with OCRL mutations [16]. The antibody was thus used throughout this study.

**Figure 1:**
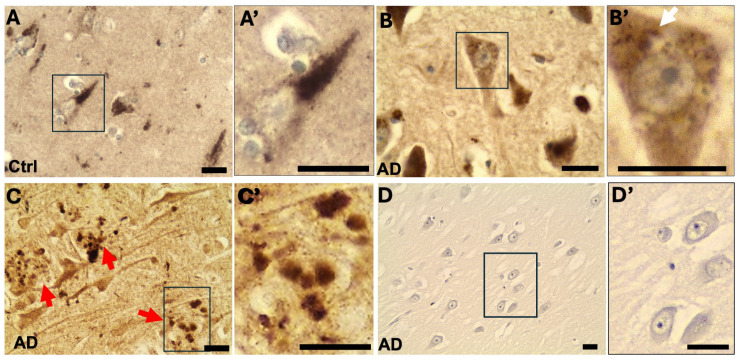
OCRL immunostaining in the CA1-2 regions of the human hippocampus. (**A**) In non-demented control brains, OCRL immunoreactivity was observed as intracellular granular structures in hippocampal pyramidal neurons. (**B**) In AD brains, a similar granular pattern was observed. In neurons exhibiting granulovacuolar degeneration (GVD), OCRL immunoreactivity was localized to the cytoplasm surrounding membrane-bound GVD structures (white arrow in (**B′**)). (**C**) In AD brains, OCRL immunoreactivity was also detected in dystrophic neurites associated with amyloid plaques (red arrows). (**D**) Negative control: immunostaining of an AD brain section processed without the primary antibody. The insets (**A′**–**D′**) show magnified views of the area outlined by rectangles. Sections were counterstained lightly with hematoxylin. Scale bars: 25 µm.

In control, non-demented hippocampal tissues, OCRL immunostaining revealed a granular intraneuronal pattern in pyramidal neurons (Figure 1A). This localization pattern is consistent with previous reports of OCRL distribution in early differentiating neurons derived from human induced pluripotent stem (iPS) cells [13].

In AD brain samples, OCRL exhibited distinct and abnormal localization patterns (Figure 1B,C). In some hippocampal pyramidal neurons, we observed large, membrane-bound vacuoles characteristic of GVD, a known marker of late-stage autophagic intermediates [17]. OCRL immunoreactivity was detected in the perivacuolar cytoplasm surrounding GVD structures but was absent from the vacuolar lumen itself (Figure 1B).

Strikingly, OCRL was also found to accumulate in plaque-associated dystrophic neurites in AD brains (Figure 1C). These structures are commonly associated with abnormal axonal and dendritic processes near amyloid plaques. Control experiments omitting the primary antibody showed no signal, confirming the specificity of the observed staining (Figure 1D).

Together, these findings indicate that OCRL is not only present in the neuronal soma under physiological conditions but also accumulates abnormally in dystrophic neurites associated with amyloid plaques and near GVD structures in AD brains.

**Figure 2:**
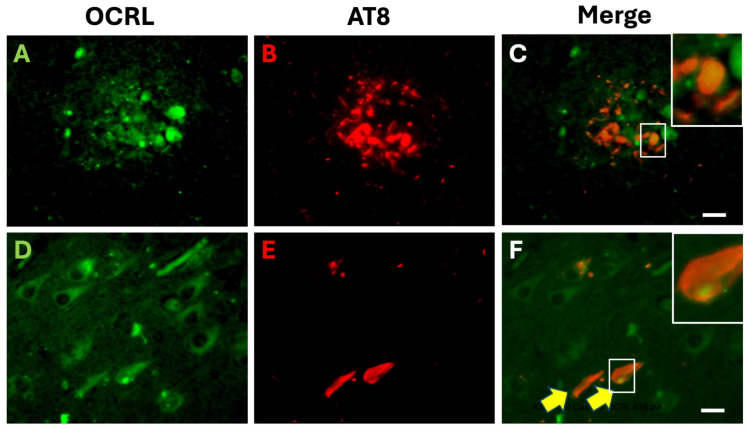
Double immunofluorescence staining of OCRL and pTau (AT8) in the hippocampus of an AD case. (**A**–**C**) Double immunofluorescence staining shows OCRL (green) and AT8-phosphorylated tau (pTau, red) in the CA1 region of the hippocampus. Merged images reveal partial colocalization of OCRL and AT8 signals in some plaque-associated dystrophic neurites. (**D**–**F**) OCRL-positive granular structures are observed in the soma of both AT8-positive (tangle-bearing, yellow arrows) and AT8-negative pyramidal neurons. Images are representative of the hippocampal CA1 region from an AD brain. The insets in (**C**,**F**) show magnified views of the area outlined by white rectangles. Scale bar, 20 µm.

To further examine the relationship between OCRL and Alzheimer’s disease pathology, we performed double immunofluorescence staining for phosphorylated tau (pTau, using the AT8 antibody) and OCRL (Figure 2). In AD brain sections, we observed partial colocalization of OCRL and AT8 in some plaque-associated dystrophic neurites (Figure 2A–C), indicating that OCRL may be present in neurites undergoing tau pathology. However, this colocalization was not complete, suggesting that OCRL may associate with a subset of tau-positive structures.

Additionally, in the hippocampal pyramidal neurons of AD brains, OCRL immunoreactivity was detected in the soma of both tangle-bearing and non-tangle-bearing neurons (Figure 2D–F), indicating that OCRL expression is not restricted to neurons with overt tau pathology.

**Figure 3:**
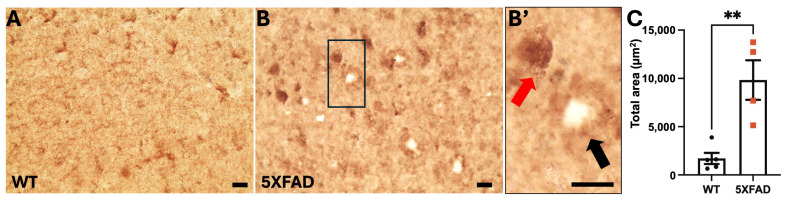
OCRL accumulates in dystrophic neurites of 5XFAD mouse brains. (**A**,**B**) Representative images of OCRL immunostaining in the cortex of 10-month-old male wild-type (WT) (**A**) or 5XFAD (**B**) mice show strong OCRL immunoreactivity in plaque-associated dystrophic neurites (black arrow in (**B′**)) and neuronal soma (red arrow in (**B′**)). The inset (**B′**) shows magnified view of the area outlined by rectangle in (**B**). (**C**) Quantification of OCRL immunolabelling by optical density analysis revealed a significant increase in the total OCRL-positive area in 5XFAD brains compared to age- and sex-matched WT controls. Data are presented as mean ± SEM (standard error of mean). Statistical significance was determined using an unpaired *t*-test (WT, *n* = 5; 5XFAD, *n* = 4). ** *p* < 0.01. Scale bar, 25 µm.

To further examine the accumulation of OCRL in plaque-associated dystrophic neurites, we analyzed OCRL immunostaining in brain sections from 5XFAD transgenic mice [18], a well-established model of amyloid pathology (Figure 3). Compared to wild-type controls, 5XFAD mice exhibited a pronounced increase in OCRL immunoreactivity (Figure 3C). The staining was primarily localized to plaque-associated dystrophic neurites, as well as to neuronal soma as observed in human AD brains.

These findings reinforce the notion that OCRL abnormally accumulates in plaque-associated dystrophic neurites in 5XFAD transgenic mouse brains, consistent with observations in human AD brain tissues.

### 2.2. RNA Levels, Protein Solubility, and Post-Translational Modifications (PTMs) of OCRL in AD Brains

#### 2.2.1. RNA Expression Levels of *OCRL* and the Autophagy Marker *BECN1* Are Not Significantly Altered in AD Brains

**Figure 4:**
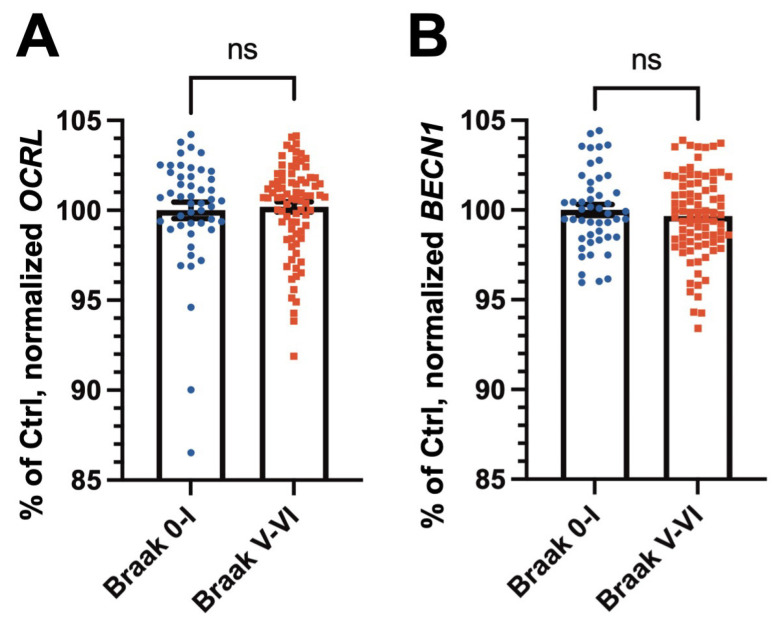
RNA expression levels of *OCRL* and *BECN1* in control and AD brains from the ROSMAP cohort. (**A**,**B**) No significant differences were observed in the transcript levels of *OCRL* (**A**) or *BECN1* (**B**) between the control and AD cases. Statistical analyses were performed on normalized datasets using the Mann–Whitney *U* test (**A**) or Student’s *t*-test (**B**) following normality assessment. ns: not significant (*p* > 0.05).

OCRL has been implicated in the regulation of autophagy, particularly in the fusion of autophagosomes with lysosomes [10]. To assess whether *OCRL* RNA expression is altered in AD, we analyzed normalized RNA expression data from the ROSMAP cohort [19]. As shown in Figure 4A, no significant difference in *OCRL* RNA levels was observed between the AD and control brain samples.

We next examined the expression of *BECN1*, which encodes Beclin1, a key component of the autophagy initiation complex [20]. Similarly, *BECN1* RNA levels showed no significant differences between the AD and control groups (Figure 4B).

To further explore these findings, we analyzed *Ocrl* and *Becn1* RNA levels in 12-month-old wild-type and 5XFAD mouse brains (Supplementary Figure S1). Consistent with the human data, no significant differences were observed for *Ocrl* and *Becn1* expression between 5XFAD mice and age- and sex-matched wild-type controls.

Together, these findings suggest that the observed dysregulation of OCRL in AD is not due to altered RNA levels. Moreover, the expression of key autophagy-related genes such as *BECN1* also remains unchanged at the RNA level in AD brains.

#### 2.2.2. OCRL Is Depleted from the RIPA-Soluble Fraction and Correlates with Beclin1 Levels in Control and AD Brains

**Figure 5:**
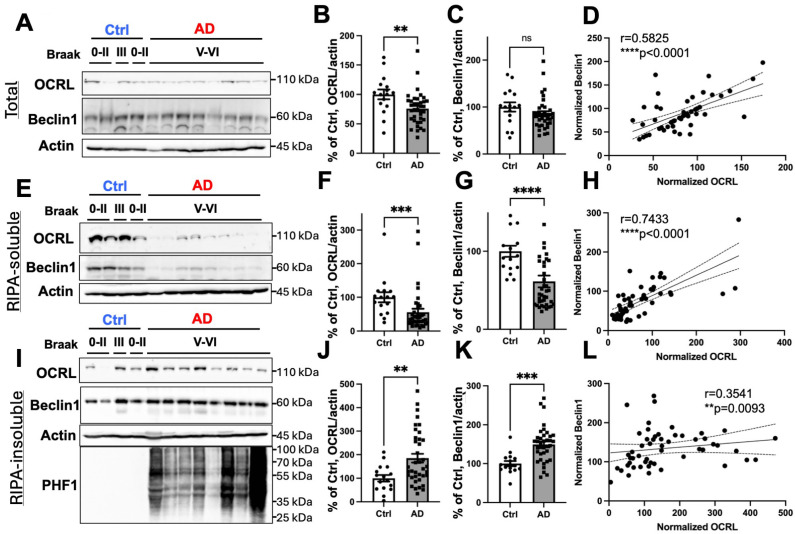
OCRL is depleted from the RIPA-soluble fraction of AD brains and correlates with Beclin1 levels. Protein levels of OCRL, Beclin1, and the loading control actin were assessed by WB in total (**A**), RIPA-soluble (**E**), and RIPA-insoluble (**I**) fractions of T1 isocortex lysates from control and AD brains. (**A**–**D**) In total lysates (**A**), OCRL levels were significantly decreased in AD brains (**B**), whereas Beclin1 levels showed no significant change (**C**). A significant positive correlation was observed between OCRL and Beclin1 in this fraction (**D**). (**E**–**H**) In the RIPA-soluble fraction (**E**), both OCRL (**F**) and Beclin1 (**G**) were significantly reduced in AD brains. Their levels were strongly and positively correlated (**H**). (**I**–**L**) In the RIPA-insoluble fraction (**I**), both OCRL (**J**) and Beclin1 (**K**) were significantly elevated in AD samples, with a corresponding significant positive correlation (**L**). The presence of phosphorylated tau was confirmed using the PHF1 antibody (**I**). Statistical analyses were performed on actin-normalized datasets using the Mann–Whitney *U* test and Spearman’s correlation. Correlation plots display the 95% confidence interval. Samples were derived from control cases (Braak stages 0–IV; *n* = 16) and AD cases (Braak stages V–VI; *n* = 38), including two familial AD (FAD) cases with *amyloid precursor protein* (*APP*) or *presenilin1* (*PSEN1*) mutations (Supplementary Table S1). Uncropped full images of WB are shown in Supplementary Figures S2–S4. ns, not significant; ** *p* < 0.01; *** *p* < 0.001; **** *p* < 0.0001.

We next examined OCRL and the autophagy marker Beclin1 protein levels by Western blotting (WB) in T1 isocortex lysates from control and AD brain samples (Figure 5). Consistent with previous reports in human cell models [13], OCRL was detected as a 110 kDa band in both the control and AD brains. Quantification revealed a significant reduction in OCRL protein levels normalized to actin in the total fraction of AD samples compared to controls (Figure 5A). In contrast, Beclin1 levels were moderately reduced in the total fraction of AD brains, but the difference did not reach statistical significance (Figure 5C).

To further explore whether OCRL solubility is altered in AD, we performed biochemical fractionation to separate RIPA-soluble and RIPA-insoluble protein fractions. In non-demented control brains, OCRL was predominantly detected in the RIPA-soluble fraction. Strikingly, in AD brains, OCRL was almost undetectable in the RIPA-soluble fraction (Figure 5E–G). Instead, it accumulated in the RIPA-insoluble fraction, which was solubilized using urea [21,22,23,24] (Figure 5I,J). These results indicate a marked shift in OCRL solubility in AD brain lysates, suggesting its aggregation or association with insoluble protein complexes.

Similarly, Beclin1, which showed no major changes in total lysates (Figure 5C), also showed a solubility shift in AD brains, being depleted from the RIPA-soluble fraction and enriched in the RIPA-insoluble fraction (Figure 5I–K). Notably, OCRL and Beclin1 levels were significantly correlated within the RIPA-soluble fraction (Figure 5H), and this positive correlation was also observed in the RIPA-insoluble fraction (Figure 5L). These findings suggest a coordinated mislocalization or potential co-aggregation of these proteins in the context of AD pathology. To confirm the pathological stage of the AD samples, we additionally assessed tau pathology using the PHF1 antibody. Consistent with clinical diagnosis, phosphorylated tau was elevated in the AD samples (Figure 5I).

In summary, OCRL is redistributed from the RIPA-soluble to the RIPA-insoluble fraction in AD brains, mirroring a similar shift observed for Beclin1.

**Figure 6:**
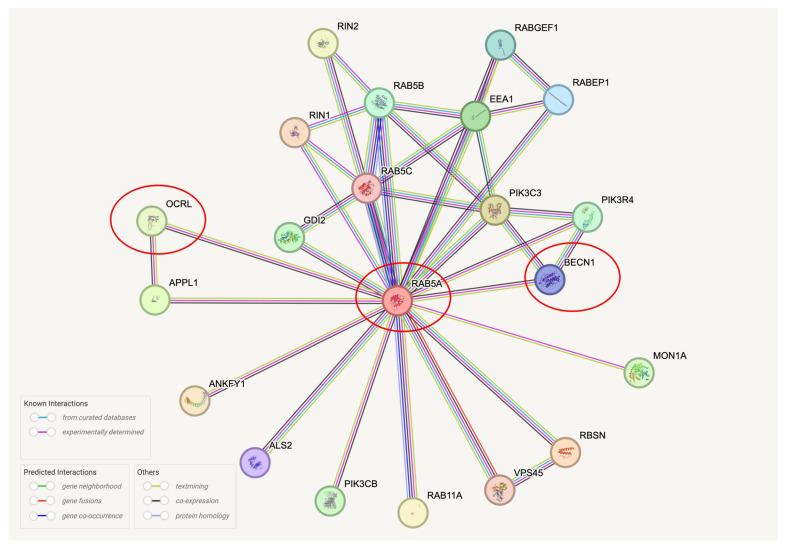
Potential indirect interaction between OCRL and Beclin1 via RAB5A. STRING analysis of protein–protein interactions suggests that OCRL and Beclin1 may be part of the same protein complex through their shared interaction with RAB5A. The analysis was performed using the STRING database (https://string-db.org/ (accessed on 1 March 2025)) on the full STRING network, with evidence-based interaction scores filtered at the highest confidence level (0.900) and a maximum of 20 proteins displayed. OCRL, Beclin1, and RAB5A are highlighted with red circles.

Although we observed concomitant misregulation of the autophagy marker Beclin1 and OCRL in AD brains, it remains unclear whether these two proteins interact directly or indirectly. To explore this, we conducted STRING analyses [25,26,27,28,29,30,31,32,33,34,35,36,37]. Current evidence does not support the formation of a direct Beclin1-OCRL protein complex. However, both proteins share a common binding partner: RAB5A (Figure 6).

#### 2.2.3. No Aberrant Post-Translational Modifications of OCRL Were Detected in AD Samples by 2D WB

Aberrant phosphorylation is a hallmark of AD pathogenesis, with several proteins, including tau [38], Pin1 [39], and others [40], undergoing significant post-translational modifications (PTMs) during disease progression. Given the pronounced solubility shift of OCRL observed in AD brains, we investigated whether OCRL might also be subject to altered PTMs using two-dimensional (2D) WB.

**Figure 7:**
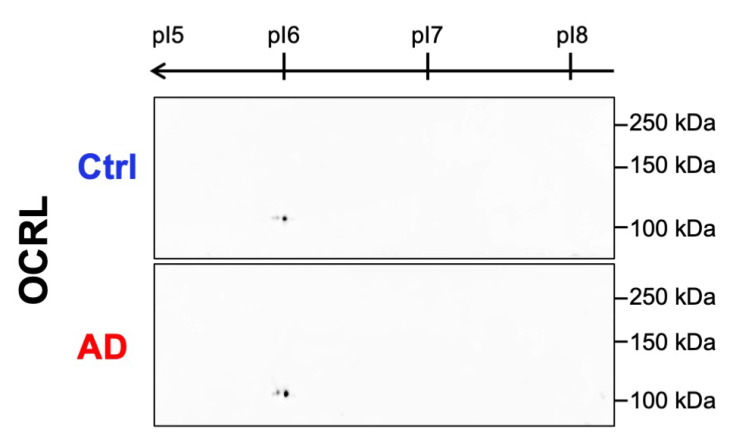
OCRL 2D migration profiles in control and AD brains. Representative 2D gel images showing the migration pattern of OCRL in brain samples from non-demented controls and AD cases. No significant differences in the overall migration profiles were observed between the two groups. Estimated isoelectric points (pI) are indicated.

OCRL has a theoretical isoelectric point (pI) of 6.13 and is known to undergo various PTMs, including ubiquitination and phosphorylation [41]. However, our 2D WB analysis revealed no detectable differences in OCRL migration pattern between the AD and control brain samples (Figure 7). The 2D profiles of OCRL in AD brains were highly similar to those in non-demented control samples, showing no evidence of aberrant pI shifts or altered spot patterns.

These results suggest that OCRL did not undergo detectable aberrant phosphorylation or PTM-related pI shifts such as acetylation under our experimental conditions. Therefore, the observed solubility change in OCRL in AD is likely mediated by mechanisms other than phosphorylation.

### 2.3. OCRL Overexpression Attenuates FRET-Positive Tau Inclusions in HEK Tau RD P301L FRET Biosensor Cells

**Figure 8:**
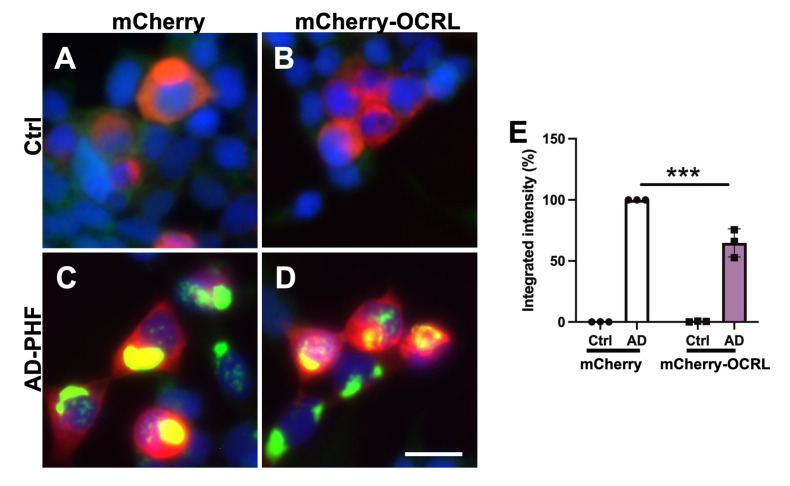
OCRL overexpression significantly attenuates FRET-positive tau oligomers in HEK tau RD P301S FRET biosensor cells transduced with AD-PHF. (**A**–**D**) Representative images of HEK Tau RD P301S FRET biosensor cells fixed 48 h after transduction. Nuclei were counterstained with DAPI. Co-transduction with the sarkosyl-insoluble fraction from a control brain and either an empty mCherry-expressing vector (**A**) or an mCherry-OCRL plasmid (**B**) did not induce FRET-positive tau inclusions. In contrast, co-transduction with AD-PHF (sarkosyl-insoluble fraction from an AD brain) and empty mCherry-expressing vector induced robust FRET-positive tau inclusions (**C**), which were significantly reduced in cells co-transduced with AD-PHF and mCherry-OCRL (**D**). (**E**) Quantification by FRET flow cytometry of 10,000 cells per condition showed a significant decrease in integrated mean fluorescence intensity in OCRL-overexpressing cells. Representative results of FACS cell sorting are shown in Supplementary Figure S5. Data represent three independent experiments. *** *p* < 0.001 by two-way ANOVA. Scale bar, 20 μm.

Given the involvement of OCRL in endocytic pathways, we hypothesized that OCRL might influence tau pathology [42]. To investigate the potential role of OCRL in modulating tau seeding, we assessed the effect of OCRL overexpression on tau–tau interactions using HEK Tau RD P301L FRET biosensor cells [43,44] (Figure 8).

The biosensor cells were co-transduced with the sarkosyl-insoluble fraction derived from either a non-demented control brain or an AD brain containing paired helical filaments (AD-PHF), along with either an empty vector (expressing mCherry) or a plasmid encoding wild-type human OCRL fused to mCherry [45]. Neither transduction with the control sarkosyl-insoluble fraction nor overexpression of OCRL alone resulted in a detectable FRET signal (Figure 8A,B).

In contrast, transduction with the AD-PHF fraction induced a robust FRET signal in control cells expressing mCherry (Figure 8C) and in cells overexpressing mCherry-OCRL (Figure 8D). However, the FRET signal was significantly lower in cells co-transduced with AD-PHF and mCherry-OCRL compared to those co-transduced with AD-PHF and mCherry alone (Figure 8E and Supplementary Figure S5A–D).

These findings suggest that OCRL overexpression reduces tau seeding and aggregation in this cellular model.

## 3. Discussion

In this study, we demonstrate the abnormal accumulation of OCRL in plaque-associated dystrophic neurites in the brains of AD patients and 5XFAD mouse model. We also provide the first evidence that OCRL undergoes a solubility shift in AD brains, becoming depleted from the RIPA-soluble fraction and enriched in the insoluble fraction, in correlation with the autophagy marker Beclin1. Despite these protein-level changes, the RNA expression levels of *OCRL* and *BECN1* remained unchanged in AD brains. Furthermore, no significant differences were observed in the global 2D migration profiles of OCRL, suggesting that aberrant hyperphosphorylation is unlikely to account for the solubility shifts. OCRL overexpression attenuated tau seeding in FRET-based cell model.

GVDs, commonly observed in pyramidal neurons of the AD hippocampus, are double-membrane-bound structures thought to represent late-stage autophagic intermediates [46]. While OCRL was not detected within the central core of GVDs, we observed OCRL immunoreactivity in the surrounding cytoplasm, suggesting a potential involvement in the autophagic process. Supporting this, OCRL levels significantly correlated with Beclin1 in post-mortem AD brains, reinforcing a link between OCRL dysregulation and impaired autophagy.

Beyond its role in phosphoinositide metabolism, OCRL regulates lysosomal positioning and trafficking. Disruption of OCRL impairs microtubule-organizing center function and lysosome localization, leading to mTORC1 inactivation, defective nutrient sensing, and upregulation of lysosomal genes [10,47]. Given the central role of lysosomes in autophagy, inter-organelle communication, and nutrient signaling, OCRL dysfunction could contribute to the enlarged lysosomes observed in AD neurons [48,49], where autophagosome–lysosome fusion is known to be impaired [20]. Similar defects have been reported in OCRL-knockdown models and in Lowe syndrome [10,50]. OCRL is recruited to lysosomes via an AP2- and clathrin-dependent mechanism during autophagosome–lysosome fusion [10]. Notably, transcriptome-wide association studies have identified significant splicing alterations in *AP2A1*, *AP2A2*, and *PICALM*, key autophagy-related genes, in AD brains [51,52]. Such alterations may contribute to the OCRL mislocalization and solubility changes, further disrupting endo-lysosomal and autophagy pathways in AD.

While OCRL protein levels and solubility were altered, RNA expression did not show significant changes. Such discrepancies between RNA and protein levels are common [53,54]. For example, a study examining over 1000 genes across multiple human cell lines found that more than 60% showed no significant correlation between mRNA and protein levels [55]. RNA-binding proteins (RBPs), protein degradation mechanisms, and sequestration into insoluble complexes may underlie this discrepancy [56,57]. Protein–protein interactions or subcellular mislocalization may also contribute to OCRL dysregulation in AD. Future proteomic studies focusing on the OCRL interactome in AD brains may clarify these mechanisms.

Although no direct interaction between OCRL and Beclin1 has been reported, the two proteins share several functional similarities. First, both interact with and are regulated by PIs, particularly PI(4,5)P_2_ and PI(3)P—lipids known to be dysregulated in AD [5,6,58,59,60,61]. Second, both proteins are involved in autophagy and membrane trafficking and interact with RAB5A. OCRL facilitates autolysosomal fusion via turnover of PI(4,5)P_2_ [10], while Beclin1 is essential for autophagosome maturation and lysosomal fusion [62].

The concurrent solubility shift may not reflect a direct interaction between OCRL and Beclin1 but rather an indirect association mediated by their shared binding partner, RAB5A, which itself undergoes pathological alterations in AD [49]. The accumulation of OCRL and Beclin1 in the insoluble fraction may indicate their involvement in pathological protein aggregation and impaired autophagy, both hallmarks of AD. More precisely, OCRL binds directly to the active, GTP-bound form of RAB5A through its ASPM-SPD2-Hydin (ASH) and RhoGAP-like domains [63,64]. RAB5A recruits OCRL to early endosomes, where OCRL hydrolyzes PI(4,5)P_2_ to facilitate proper endosomal maturation [65,66]. RAB5A, a key marker of early endosomes, plays a central role in regulating endocytic vesicle trafficking and fusion [67]. Notably, RAB5-positive early endosomes are enlarged in neurons affected by AD [49,68], and dysfunction of RAB5-mediated endocytosis has been implicated in the early stages of AD pathogenesis [69]. Given OCRL’s role in endosomal sorting and trafficking to lysosomes, its disruption could impair Beclin1-dependent autophagosome–lysosome fusion. Moreover, Beclin1 can activate RAB5A to promote the endosomal degradation of autophagic substrates [70,71]. These shared pathways suggest the potential for functional or indirect associations between OCRL and Beclin1, possibly mediated by RAB5A or other shared interacting partners.

Functionally, OCRL overexpression significantly reduced tau seeding in HEK tau RD P301L FRET biosensor cells transduced with AD-PHF, suggesting a protective role in development of tau pathology. Loss of OCRL function is known to cause intracellular accumulation of PI(4,5)P_2_, as observed in cells from Lowe syndrome patients [72], and similar accumulation has been implicated in tau pathology in AD brains [4,73]. As a PI 5-phosphatase, OCRL may mitigate tau seeding by reducing PI(4,5)P_2_ levels at the plasma membrane. Since clathrin-mediated endocytosis is a major route for cellular uptake of tau seeds [74,75,76], reduced PI(4,5)P_2_ may impair the process and limit tau internalization. Additionally, OCRL may promote endolysosomal maturation and autophagic degradation of tau aggregates, facilitating their clearance and preventing intracellular propagation. The functional association with RAB5A also links OCRL to early endosomal trafficking, a process implicated in tau hyperphosphorylation and neurodegeneration [77]. Furthermore, OCRL is involved in maintaining endosomal membrane integrity [78], and thus dysregulation of OCRL may well be involved in the endolysosomal escape of tau aggregates for cytosolic tau seeding [79]. Collectively, these findings suggest that OCRL dysfunction, possibly in conjunction with other PI 5′-phosphatases such as Synaptojanin 1 and SHIP2 [6,22,80], may contribute to phosphoinositide imbalance and downstream pathological processes in AD. Given the observed depletion of OCRL from soluble fractions in AD brains, restoring its expression may represent a promising therapeutic strategy to reduce tau aggregation and propagation.

However, several limitations of this study should be acknowledged. First, although a correlation between OCRL expression and reduced Beclin1 levels was observed, we did not directly demonstrate that OCRL dysfunction leads to impaired autophagy in an AD model. Thus, whether OCRL dysregulation causally contributes to AD pathogenesis remains unresolved. In vivo studies using transgenic mouse models with OCRL overexpression or knockdown in the context of amyloid or tau pathology will be essential to address this question. Additional analyses investigating OCRL in relation to RAB5A would also be informative. Second, although 2D WB did not reveal shifts in pI of OCRL, this does not exclude the presence of other PTMs such as oxidation, methylation, or crosslinking. Mass-spectrometry-based analyses will be essential to further characterize these potential modifications. Finally, although OCRL overexpression attenuated tau seeding in vitro, the underlying molecular mechanism remains to be elucidated.

Despite these limitations, our study provides novel and valuable insights into the involvement of OCRL in AD pathology. The evidence presented here supports a role for OCRL in modulating tau pathology and highlights its interactions with autophagic processes. These findings may pave the way for future therapeutic strategies targeting OCRL or its associated pathways to slow or prevent AD progression.

## 4. Materials and Methods

### 4.1. Antibodies

Rabbit polyclonal anti-OCRL antibodies were purchased from Proteintech (fisher scientific, Merelbeke, Belgium, 17695-1-AP) raised against 538–893 amino acids of OCRL encoded by BC094726. This antibody reacts with both human and mouse OCRL. Mouse monoclonal anti-actin (A-5441) and rabbit polyclonal anti-GAPDH (G-4644) antibodies were purchased from Sigma-Merck, Hoeilaart, Belgium. Mouse monoclonal anti-phospho-Tau (Ser202, Thr205) AT8 antibody was purchased from Thermo Fisher Scientific, Zaventem, Belgium (Catalog # MN1020). Mouse monoclonal PHF1 antibody was provided by Dr. Peter Davies and recognizes pSer396/404 of tau [81]. Rabbit polyclonal anti-Beclin1 (H-300) was purchased from Santa Cruz Biotechnology, Heidelberg, Germany (sc-11427).

### 4.2. Human Brain Tissues

Frozen samples from the superior temporal T1 isocortex or formalin-fixed hippocampal tissue were obtained from individuals with AD and age-matched non-demented control subjects. Control cases were defined as individuals without dementia who died without any known neurological disorders. AD cases were diagnosed according to the National Institute of Aging and Reagan Institute Criteria [82] and scored by neuropathological staging for tau and amyloid pathologies [83,84]. AD cases, including two familial AD (FAD) cases with *amyloid precursor protein* (*APP*) or *presenilin1* (*PSEN1*) mutations, were all scored as Braak’s stage V or VI (Supplementary Table S1). The mean ages and post-mortem delays of control cases and of AD patients were not significantly different. Average age at death was 72.68 +/− 12.06 and 75.55 +/− 10.97 years for control (*n* = 22) and AD (*n* = 44) cases, respectively (mean +/− SD (standard deviation)) (*p* = 0.3372). Average post-mortem delays were 21.54 +/− 12.33 h and 23.65 +/− 13.75 h for control and AD cases (mean +/− SD) (*p* = 0.6135). *Apolipoprotein E* (*APOE*) genotype was determined for cases with an informed consent for genetic study using PCR (polymerase chain reaction) amplification for genomic DNA and sequencing as described [85].

### 4.3. Preparation of Brain Homogenates for Biochemical Analysis

About 200 mg of frozen T1 isocortex was homogenized, as reported in [86,87], in 10 volumes of ice-cold RIPA buffer containing 50 mM Tris-HCl pH 7.4, 50 mM NaCl, 1% NP-40, 0.25% sodium deoxycholate, 5 mM EDTA, 1 mM EGTA, complete protease inhibitor cocktail (Sigma-Merck, Hoeilaart, Belgium, 11697498001), 1 mM PMSF (Sigma-Merck, Hoeilaart, Belgium, P-7626), and phosphatase inhibitor cocktail 2 (Sigma-Merck, Hoeilaart, Belgium, P-5726) and incubated for 60 min at 4 °C on a rotator. A total of 100 µL of the total homogenate was supplemented with Laemmli sample buffer, sonicated on ice, and analyzed as the total fraction. The rest of the total homogenate was centrifuged (20,000× *g* for 20 min at 4 °C), and the supernatant was used as a RIPA-soluble fraction. The RIPA-insoluble pellet was sonicated on ice (10 pulses of 1 sec with 1 sec interval) in a 5-fold volume of 8 M urea containing protease and phosphatase inhibitors and incubated for 30 min at room temperature on a rotator. The mixture was centrifuged at 20,000× *g* at 4 °C for 30 min. The supernatant was used as a RIPA-insoluble fraction. For each fraction, protein concentrations were estimated by the Bradford method (Bio-Rad, Lokeren, Belgium, 5000205) before addition of Laemmli sample buffer. A total of 25 µg of protein was loaded to each well for SDS-PAGE.

For 2D WB analyses, post-mortem brain samples were homogenized and denatured in a buffer containing 7 M urea and 2 M thiourea. The samples were analyzed using 5–8 IPG strips (Bio-Rad, Lokeren, Belgium) as previously described [88].

### 4.4. Preparation of Human Sarkosyl Insoluble PHF-Tau Fraction

Sarkosyl fractionation of human brain tissue was carried out as previously described [89,90,91]. A total of 0.5 g of frozen frontal cortex from control (Braak I, Thal 0) and AD (Braak V–VI, Thal 4) cases was homogenized in 10 volumes of ice-cold PHF-extraction buffer (10 mM Tris-HCl (pH 7.4), 0.8 M NaCl, 1 mM EDTA, 10% sucrose). The homogenate was centrifuged at 15,000× *g* for 20 min at 4 °C. N-lauroylsarcosine sodium salt (L-5125; Sigma-Merck, Hoeilaart, Belgium) was added to the supernatant to reach a final concentration of 1% (*w*/*v*). The lysate was incubated overnight with a mild agitation at 4 °C followed by an ultracentrifugation at 180,000× *g* for 30 min at 4 °C. The Sarkosyl-soluble supernatant was removed, and the Sarkosyl-insoluble pellet was briefly rinsed and re-suspended in 0.25 mL of PBS (pH 7.4) by vigorous pipetting. The protein concentration was determined by a Bradford protein assay (Bio-Rad, Lokeren, Belgium) and adjusted to 1 mg/mL. This Sarkosyl-insoluble PHF-tau fraction was aliquoted and kept at −80 °C. Sarkosyl-insoluble fractions were analyzed by WB and transmission electron microscopy as previously described [22,86].

### 4.5. Analyses of RNA Expressions Human and Mouse Data Sets

For human datasets, the normalized RNA expression data were obtained from Rush University via the data depository (https://www.synapse.org/Synapse:syn3800853 (accessed on 1 December 2023)) [92]. The Religious Orders Study (ROS) and Rush Memory and Aging Project (MAP) are both prospective cohort studies of aging and dementia. Microarray data were obtained from the dorsolateral prefrontal cortex (PFC) of 490 samples without replicates [93]. They used an Rneasy lipid tissue kit (Qiagen, Valencia, CA, USA) for RNA extraction and an Illumina^®^ TotalPrepTM RNA Amplification Kit from Ambion, Inc (Austin, TX, USA). (catalogue # L-1755) for label protocol. These data include sex, race, age of death, *APOE* genotypes, and clinical and neuropathological scores (Braak and CERAD standing for Consortium to Establish a Registry for AD). All participants were non-demented at enrolment and had annual clinical assessments. At death, brains went for a quantitative neuropathologic assessment [19]. The data were analyzed according to neuropathological Braak staging of tau [83]. 

For mouse datasets, the normalized RNA expression of *Ocrl* and *Becn1* was analyzed using publicly available RNA-seq datasets from the Jax.IU.Pitt_5XFAD study (https://www.synapse.org/Synapse:syn22323073 (accessed on 1 March 2025)).

### 4.6. WB

Tissue lysates were run in 7.5% Tris–glycine gels and transferred onto nitrocellulose membranes (sc-3724, Santa Cruz Biotechnology, Heidelberg, Germany). The nitrocellulose membranes were blocked in 10% (*w*/*v*) semi-fat dry milk in TBS (Tris-HCl 0.01 M, NaCl 0.15 M, pH 7.4) for 1 h at room temperature and were incubated with primary antibodies overnight followed by rinses and an incubation with anti-rabbit (#7074, Cell Signaling Technology, Bioké, Leiden, The Netherlands) or anti-mouse (A-6782, Sigma-Merck, Hoeilaart, Belgium) immunoglobulin conjugated to horseradish peroxidase. After several rinses, the membranes were incubated with SuperSignal West Pico PLUS Chemiluminescent Substrate (Pierce, Thermo Fisher Scientific, Zaventem, Belgium) and were exposed to a DARQ-7 CCD cooled camera (Vilber-Lourmat, Marne-la-Vallée, France) in a SOLO 4S WL system (system version of 2014). Levels of optical density (OD) of protein signals were estimated by densitometry analysis using the NIH ImageJ program (Version 1.53a). OD of the actin signal was used to normalize protein loading.

### 4.7. Mouse Lines

The 5XFAD double transgenic mice (Tg6799 line) were provided by Dr. Robert Vassar (Northwestern University, Chicago, IL, USA). These mice co-express and co-inherit the 695 amino acids isoform of the human *APP* (APP695) carrying the Swedish, Florida, and London mutations and the human *PSEN1* carrying the M146L and L286V mutations (Tg6799 line) [18]. Genotyping was performed by PCR amplification of genomic DNA as reported previously [24]. The line was maintained on a C57BL6J background. Mice were sacrificed at 10 months by cervical dislocation without anesthesia, and brains were dissected. Brains were fixed for 24 h in 10% formalin for histological analysis. All animal studies were performed in compliance with the ethical guidelines and approved by the Ethical Committee for the Care and Use of Laboratory Animals at the Medical School of the Free University of Brussels.

### 4.8. Immunohistochemistry

After formaldehyde fixation (10% buffered formalin), brain tissues were paraffin-embedded and sliced in 7 µm thick sections. Staining by 3, 3′-diaminobenzidine (DAB) was performed as previously described [87], and the sections were observed with a Leica DM500 microscope. Double immunofluorescence labelling was performed as previously reported [94]. Mouse monoclonal AT8 antibody was used at 1/100 and detected with a donkey anti-mouse antibody conjugated with Alexa488 (A21206, Invitrogen, Thermo Fisher Scientific, Zaventem, Belgium). Rabbit polyclonal anti-OCRL antibody was used at 1/100 and detected using a biotin-labelled secondary antibody (A16027, Invitrogen, Thermo Fisher Scientific, Zaventem, Belgium) followed by incubation with Streptavidin-Alexa594 (S11227, Invitrogen, Thermo Fisher Scientific, Zaventem, Belgium). Slides were counterstained with DAPI and mounted with Glycergel (Dako, Agilent, Machelen, Belgium). Immunofluorescence labelling was observed with an Axiovert 200 M microscope equipped with an ApoTome system (Zeiss, Zaventem, Belgium).

For quantitative analysis, OCRL-positive structures in the cortex of wild-type and 5XFAD mice were analyzed at 40× images by thresholding analyses using NIH ImageJ (Version 1.53a) as previously reported [95,96].

### 4.9. Cell Culture

Tau RD P301S FRET Biosensor cells (CRL-3275) were purchased from ATCC (LGC, Molsheim, France). This cell line was derived by transducing HEK293T cells with 2 separate lentivirus constructs encoding tau RD P301S-CFP and tau RD P301S-YFP [44]. The cells were cultured in Dulbecco’s Modified Eagle’s Medium (DMEM) supplemented with 10% fetal bovine serum (FBS), 100 I.U./mL penicillin/streptomycin and 2 mM L-Glutamine (Gibco, Thermo Fisher Scientific, Zaventem, Belgium) in a humidified incubator at 37 °C with 5% CO_2_.

### 4.10. Liposome-Mediated Transduction of Sarkosyl Insoluble Fraction Containing AD-PHF in Tau RD P301S FRET Biosensor Cells

Tau RD P301S FRET Biosensor cells were plated at a density of 40,000 cells per well in a 6-well plate. Twenty-four hours later, at 60% confluency, the cells were transduced with plasmid (an empty vector expressing mCherry alone or mCherry-tagged human wild-type OCRL) [10,45]. Transduction complexes were made by combining 1 µg of plasmid, 1 µg of sarkosyl-insoluble fraction (control or AD-PHF), and 2 µL of jetPRIME (Polyplus, VWR, Leuven, Belgium) reagent with jetPRIME buffer for a total volume of 100 μL per well, as previously described [43]. Liposome preparations were incubated at room temperature for 10 min before adding to cells cultured in 1 mL of complete medium per well. Cells were incubated with transduction complexes for 48 h before harvesting.

### 4.11. Fluorescence-Activated Cell Sorting (FACS)

For fluorescence-activated cell sorting (FACS), the cells in 6-well plates were harvested with 300 µL of 0.05% Trypsin-EDTA (Gibco), mixed with 1 mL complete medium, and centrifuged at 1000× *g* for 5 min to make a cell pellet. The collected cells were re-suspended in 500 µL of sterile PBS containing 2% FBS and analyzed for FRET flow cytometry (BD LSRFortessa™ X-20 Cell Analyzer, BD Biosciences, Erembodegem, Belgium). mCherry-positive cells were first collected by flow cytometry prior to analyzing the FRET signal. The integrated mean fluorescence intensity of was normalized to that of control cells co-transduced with AD-PHF and empty vector encoding only mCherry [97].

### 4.12. Fixation and Counterstaining of Tau RD P301S FRET Biosensor Cells

For fluorescent microscopy analyses, the cells on coverslips were rinsed three times with PBS and were fixed for 10 min with 4% paraformaldehyde (PFA) in PBS containing 4% sucrose. After the fixation, the cells were rinsed in TBS, and the cells were incubated with DAPI for 30 min at room temperature. After three rinses with TBS, the cells were mounted in Glycergel (Dako).

### 4.13. Statistical Analyses

The number of samples is indicated in the figure legends. Statistical analyses and normality tests were performed using GraphPad Prism 9. Comparisons were conducted using Student’s unpaired two-tailed *t*-tests for parametric data, Mann–Whitney’s *U* tests for non-parametric data, or one-way ANOVA, as specified in the figure legends. Data are presented as mean ± SEM. *p*-values < 0.05 were considered statistically significant.

## 5. Conclusions

In this study, we showed the alteration of the protein localization of PI 5′-phosphatase OCRL in AD brains. In correlation with autophagy marker Beclin1, OCRL was depleted from the soluble fraction of AD brain lysates. Using HEK tau biosensor cells, we observed a protective effect of OCRL overexpression in tau seeding. Our data provide an insight into the potential involvement of OCRL in the progression of AD.

## Data Availability

The original contributions presented in this study are included in the article/Supplementary Material. Further inquiries can be directed to the corresponding authors. Full-length uncropped WB images are available in Supplementary Figures.

## Supplementary Materials

The following supporting information can be downloaded at: https://www.mdpi.com/article/10.3390/ijms26125827/s1.
