# Supplementary material for: Dysregulation of Inositol Polyphosphate 5-Phosphatase OCRL in Alzheimer’s Disease: Implications for Autophagy Dysfunction
Source: Int J Mol Sci. 2025 Jun 18;26(12):5827. doi: 10.3390/ijms26125827 (PMC12192959; doi:10.3390/ijms26125827)
Supplement: Supplementary file 1 [file ijms-26-05827-s001.zip › ijms-3658668-supplementary.pdf]

Supplementary information

Case #	Clinical diagnostics	Braak	Thal	Sex	Age	PMD	ApoE	Analyses
No. 1	Control	0	0	M	69	6	E2/E3	WB
No. 2	Control	II	0	M	79	NA	E2/E3	WB
No. 3	Control	0	0	F	60	28	E3/E3	WB
No. 4	Control	0	NA	F	86	NA	NA	WB
No. 5	Control	II	0	M	73	10	E3/E3	WB
No. 6	Control	0	1	M	84	13.5	E3/E3	WB
No. 7	Control	I	0	F	92	NA	NA	WB
No. 8	Control	III	0	M	58	5.5	NA	WB
No. 9	Control	0	NA	M	72	24	E3/E3	WB
No. 10	Control	0-I	NA	M	81	16.5	E3/E3	WB, IHC
No. 11	Control	I	NA	F	89	35	E2/E4	WB
No. 12	Control	I-II	0	M	67	24	E3/E3	WB, IHC
No. 13	Control	0	0	F	43	NA	NA	IHC
No. 14	Control	II	0	M	80	8	NA	IHC
No. 15	Control	I	0	F	57	24	NA	IHC
No. 16	Control	I	0	M	65	NA	NA	IHC
No. 17	Control	0	0	M	63	NA	NA	IHC
No. 18	Control	1	4	M	69	NA	NA	IHC
No. 19	Control	III	4	F	82	NA	E3/E3	WB
No. 20	Control	III	NA	M	70	31	E3/E3	WB
No. 21	Control	IV	2	F	76	28	E3/E3	WB
No. 22	Control	IV	1	M	84	48	E3/E3	WB
No. 23	AD	VI	4	M	60	37	E3/E3	WB
No. 24	AD	VI	NA	M	67	19	E3/E4	WB
No. 25	AD	VI	NA	M	57	19	E3/E4	WB
No. 26	AD	VI	4	M	79	28	E3/E4	WB
No. 27	AD	VI	NA	M	63	NA	NA	WB
No. 28	AD	VI	NA	M	74	10	E3/E3	WB
No. 29	AD	VI	NA	M	64	3	E3/E3	WB
No. 30	AD	VI	NA	F	89	10	E2/E3	WB
No. 31	AD	VI	NA	F	87	23	E2/E3	WB
No. 32	AD	VI	5	F	86	30	E3/E3	WB
No. 33	AD	VI	NA	M	71	6	E3/E3	WB
No. 34	AD	VI	NA	F	90	32	E2/E4	WB
No. 35	AD	VI	3	M	61	24	E3/E4	WB
No. 36	AD	VI	4	F	79	24	E3/E4	WB
No. 37	AD	VI	5	M	82	25	E3/E4	WB
No. 38	AD	VI	5	F	83	24.5	E3/E4	WB
No. 39	AD	VI	4	M	83	21	E3/E4	WB
No. 40	AD	VI	NA	M	76	9.5	E3/E4	WB
No. 41	AD	VI	4	F	80	24	E3/E4	WB

No. 42	AD	VI	NA	M	66	9.5	NA	WB
No. 43	AD	VI	NA	M	81	20	E2/E3	WB
No. 44	AD	VI	NA	F	82	20.5	E3/E3	WB
No. 45	AD	VI	5	M	84	NA	E3/E3	WB
No. 46	AD	VI	NA	F	91	26	E3/E3	WB
No. 47	AD	VI	5	F	83	24	E3/E3	WB
No. 48	AD	VI	6	M	81	17.5	E3/E3	WB
No. 49	AD	VI	NA	F	63	28	E3/E4	WB
No. 50	AD	VI	5	F	92	60	E3/E4	WB
No. 51	AD	VI	5	M	76	10	E3/E4	WB
No. 52	AD	VI	5	M	73	45	E3/E4	WB
No. 53	AD	VI	4	M	83	34	E3/E4	WB
No. 54	AD	VI	NA	M	70	6	E4/E4	WB
No. 55	AD	VI	5	F	92	60	E3/E4	WB
No. 56	AD	VI	5	M	76	10	E3/E4	WB
No. 57	AD	VI	5	M	73	45	E3/E4	WB
No. 58	AD	VI	4	M	83	34	E3/E4	WB
No. 59	FAD (*APP* G2149A)	VI	5	F	56	NA	E3/E3	WB
No. 60	FAD (*PSEN1* R35E, E120D)	VI	5	F	49	14.5	E3/E4	WB
No. 61	AD	VI	NA	F	81	8	E3/E3	IHC
No. 62	AD	VI	4	F	72	24	E3/E3	IHC
No. 63	AD	VI	4	F	70	45	E3/E4	IHC
No. 64	AD	VI	4	F	60	24	E3/E3	IHC
No. 65	AD	VI	2	F	91	5.5	E3/E4	IHC
No. 66	AD	VI	5	M	65	30	E3/E3	IHC

Table S1. Human cases analyzed in this study.

The neuropathological staging of AD patients is determined according to Braak staging for NFT scores [1] and to Thal staging for amyloid plaque scores [2]. AD: Alzheimer Disease. FAD: familial Alzheimer disease. *APP*: *Amyloid precursor protein. PSEN1*: *Presenilin1*. PMD: *post-mortem* delay. NA: not available. Frozen tissues of T1 isocortex were analyzed by western blotting (WB). Paraffin embedded tissues of T1 isocortex and hippocampus were analyzed by immunohistochemistry (IHC). *APOE* genotyping was determined only for the cases with informed consent for genomic analyses.

Supplementary figure 1

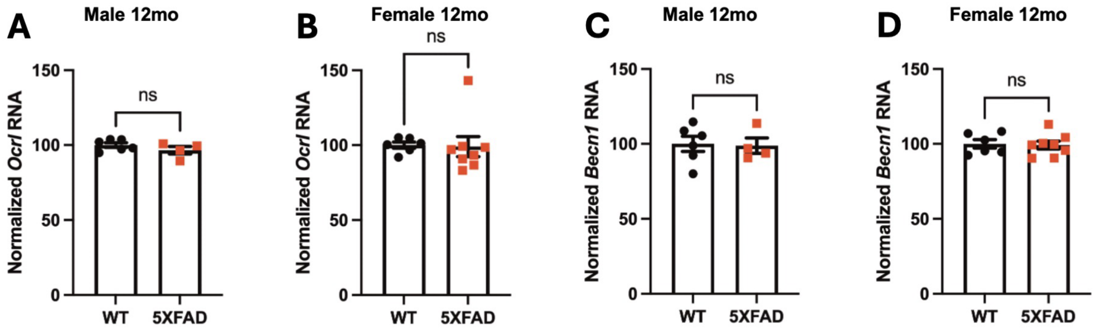

Supplementary figure S1. No change in RNA expression of *Ocrl* and *Becn1* in 12-month-old 5XFAD mouse brains. RNA expression levels of *Ocrl* and *Becn1* were analyzed in 12-month-old 5XFAD mouse brains using publicly available RNA-seq data from the Jax.IU.Pitt_5XFAD study (https://www.synapse.org/Synapse:syn22323073). No significant differences in normalized RNA levels of *Ocrl* and *Becn1* were observed between wild-type and 5XFAD mice of either sex. RNA expression was normalized to transcripts per million (TPM), and values are expressed relative to age-matched wild-type mice (set to 100%). WT: wild-type.

Supplementary figure 2

Uncropped WB images of Figure 5A (Total fraction)

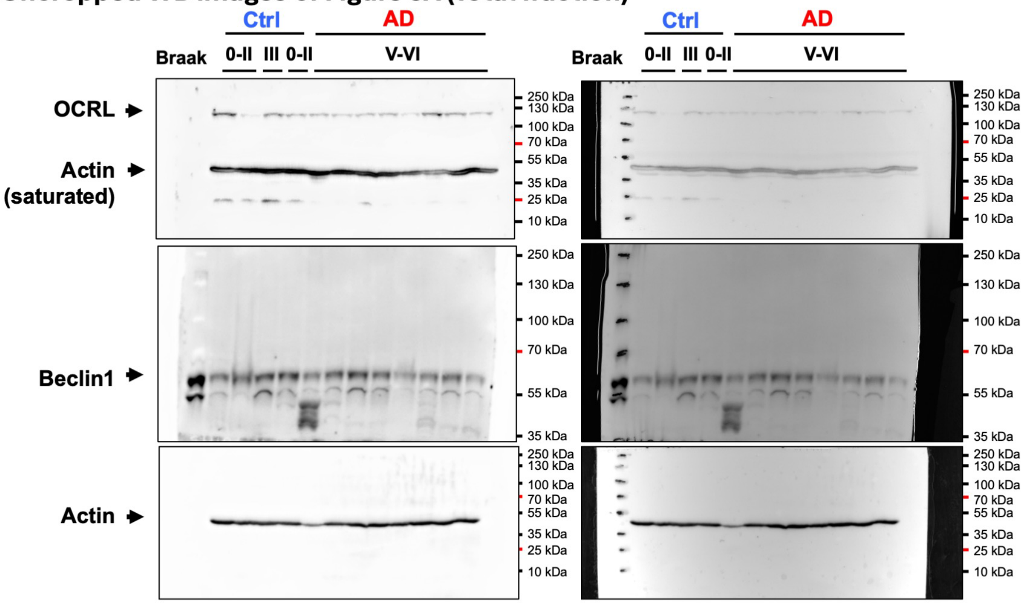

Supplementary figure S2. Uncropped full images of western blot (WB) data shown in Figure 5A. The total brain lysates were analyzed for OCRL, Beclin1 and actin. The right panels represent the merged images of the molecular weight marker (PageRuler, Thermo Scientific) and the chemiluminescence signals.

Supplementary figure 3

Uncropped WB images of Figure 5E (RIPA-soluble fraction)

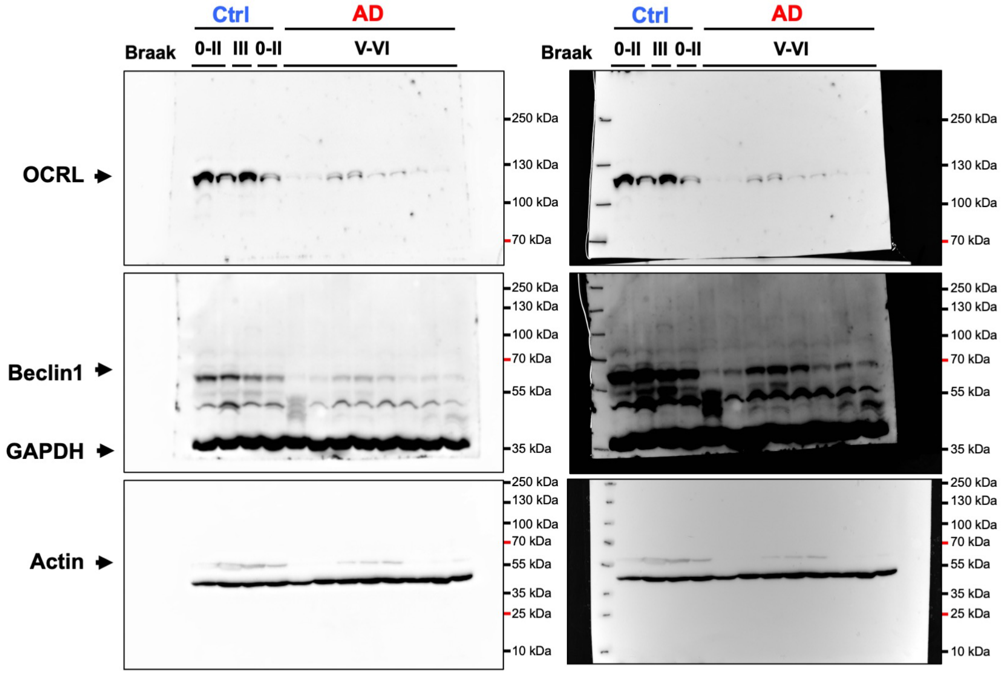

Supplementary figure S3. Uncropped full images of WB data shown in Figure 5E. RIPA-soluble fractions were analyzed for OCRL, Beclin1 and actin. The right panels represent the merged images of the molecular weight marker (PageRuler) and the chemiluminescence signals.

Supplementary figure 4

Uncropped WB images of Figure 5I (RIPA-insoluble fraction)

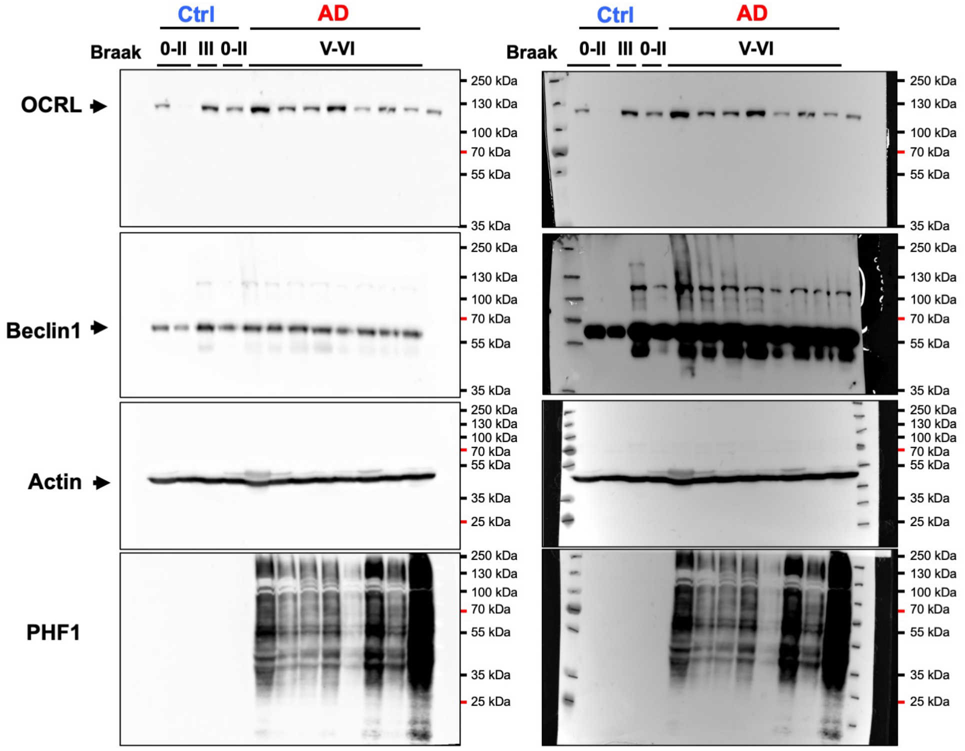

Supplementary figure S4. Uncropped full images of WB data shown in Figure 5I. RIPA-insoluble fractions were analyzed for OCRL, Beclin1 and actin. The right panels represent the merged images of the molecular weight marker (PageRuler or the Rainbow Molecular Weight Marker, Sigma) and the chemiluminescence signals.

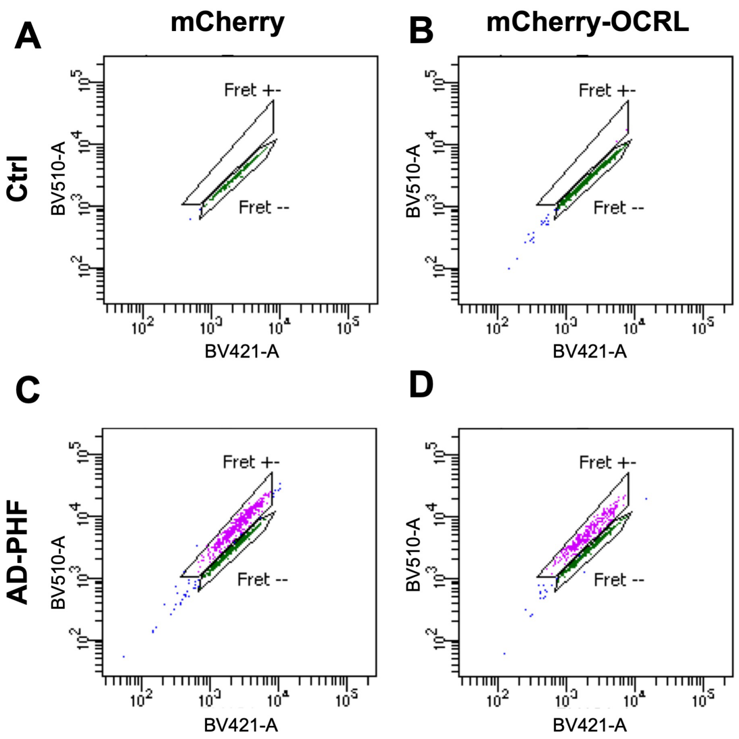

Supplementary Figure S5. OCRL overexpression significantly reduces FRET-positive tau oligomers in HEK tau RD P301S FRET biosensor cells transduced with AD-PHF.

(**A–D**) Representative FRET flow cytometry images from 10,000 cells per condition. A majority of cells remained FRET-negative (green) when co-transduced with the Sarkosyl-insoluble fraction from a control, non-demented brain and either an empty control vector expressing mCherry alone (**A**) or mCherry-OCRL (**B**). In contrast, transduction with AD-PHF led to a marked increase in FRET-positive cells (magenta) in both conditions: with mCherry alone (**C**) and mCherry-OCRL (**D**). Notably, co-transduction with AD-PHF and mCherry-OCRL resulted in a reduced FRET signal compared to AD-PHF and mCherry alone, indicating that OCRL overexpression attenuates tau oligomerization.

References

1 Braak H, Braak E (1991) Neuropathological stageing of Alzheimer-related changes. Acta Neuropathol 82: 239-259 Doi 10.1007/BF00308809

2 Thal DR, Rub U, Orantes M, Braak H (2002) Phases of A beta-deposition in the human brain and its relevance for the development of AD. Neurology 58: 1791-1800
